# Karrikinolide1 (KAR_1_), a Bioactive Compound from Smoke, Improves the Germination of Morphologically Dormant *Apium graveolens* L. Seeds by Reducing Indole-3-Acetic Acid (IAA) Levels

**DOI:** 10.3390/plants13152096

**Published:** 2024-07-29

**Authors:** Shubhpriya Gupta, Jakub Hrdlička, Manoj Kulkarni, Ivana Doležalova, Aleš Pěnčík, Johannes Van Staden, Ondřej Novák, Karel Doležal

**Affiliations:** 1 Laboratory of Growth Regulators, Faculty of Science, Palacký University & Institute of Experimental Botany AS CR, v.v.i, Šlechtitelů 11, 78371 Olomouc, Czech Republic; jakub.hrdlicka@centrum.cz (J.H.); idolezalova@vurv.cz (I.D.); alespencik@seznam.cz (A.P.); ondrej.novak@upol.cz (O.N.); karel.dolezal@upol.cz (K.D.); 2 Research Centre for Plant Growth and Development, School of Life Sciences, University of KwaZulu-Natal Pietermaritzburg, Private Bag X01, Scottsville 3209, South Africa; kulkarnim1@ukzn.ac.za (M.K.); rcpgd@ukzn.ac.za (J.V.S.); 3 Department of Chemical Biology, Faculty of Science, Palacký University, Šlechtitelů 11, 78371 Olomouc, Czech Republic; 4 Department of Genetic Resources for Vegetables, Medicinal and Special Plants, Crop Research Institute, Šlechtitelů 29, 78371 Olomouc, Czech Republic

**Keywords:** smoke-water, Karrikinolide, biostimulants, celery, phytohormones

## Abstract

Smoke-water (SW) and Karrikinolide1 (KAR_1_) release dormancy and improve seed germination in many plant species. Therefore, we tested SW (1:2500 *v*/*v*) and KAR_1_ (10^−7^ M) to break the morphological dormancy of celery cultivar (*Apium graveolens* L.). In the first trial, seeds were subjected to a 21-day incubation period at 20 °C with SW and KAR_1_ applied as single treatments. KAR_1_ showed significantly improved germination (30.7%) as compared to SW (17.2%) and a water control (14.7%). In seed soaking experiments, SW, KAR_1_, and gibberellic acid (GA_3_) treatments showed higher germination percentages than the water control after 3 and 6 h of soaking. However, prolonged soaking (12 h) reduced germination percentages for all treatments, indicating a detrimental effect. Analysis of KAR_1_ content dynamics in 7-day- and 21-day-old celery seeds indicated its prolonged effects on germination and dormancy alleviation. Phytohormones, including auxins in 7-day-old and cytokinins in 7-day- and 21-day-old celery seedlings, along with their precursors and metabolites, were analyzed using ultra-high-performance liquid chromatography-tandem mass spectrometry (UHPLC-MS/MS) after treatment with KAR_1_ and SW. The analysis of auxin levels in 7-day-old seeds revealed a negative correlation between seed germination and auxin (indole-3-acetic acid, IAA) content. Notably, it was found that KAR_1_-treated seeds significantly reduced IAA levels in all treatments. SW and KAR_1_ did not significantly affect cytokinin levels during celery germination except for N6-Isopentenyladenine. Hence, further research is needed to understand their precise role in celery seed germination. This work will improve our understanding of the role of bioactive compounds from plant-derived smoke and how they regulate hormonal responses and improve germination efficiency in celery.

## 1. Introduction

Seed germination is a critical phase in the life cycle of plants, and its success is influenced by a myriad of factors, including environmental conditions, seed quality, and physiological dormancy [1]. *Apium graveolens* (celery) is a valuable horticultural crop grown extensively for both culinary and medicinal purposes. However, the seeds of this plant species exhibit notable dormancy. Several reasons have been reported that cause dormancy in celery seeds, one of the main causes being unsuitable temperature and light conditions [2,3]. Celery seeds require an optimum temperature of 20 °C or less. Underdeveloped (small) embryos embedded in endosperm tissue also hinder celery seed germination under normal conditions [4,5]. Furthermore, the chemicals found in celery seeds, such as coumarins, may also prevent germination by leaching out of the seed coat [6,7]. Celery seeds are small in size and may take up to 21 days to germinate. They have immature embryos surrounded by hard seed coats and often do not germinate even in the presence of favorable conditions of water, oxygen, and temperature. The hard seed coat (as the pericarp is made of a double layer of testa and endosperm) of celery does not allow water to be imbibed by the seeds, preventing the initiation of physiochemical transformation in seeds [8]. Overcoming this dormancy is essential for the successful cultivation of celery. Internal and external factors are involved in coordinating the dormancy release process [9]. Internal factors mainly include phytohormones such as auxins, cytokinins, gibberellin (GA), and abscisic acid (ABA). On the other hand, external factors are environmental conditions such as light, temperature, and water. Biostimulants made from plant-derived smoke can be a promising way to release the dormancy of celery seeds.

Plant-derived aerosol smoke and smoke-water (SW) have gained popularity as a sustainable source of biostimulants that can improve seed germination and plant growth [10,11]. Karrikinolides [such as Karrikinolide1 (KAR_1_) and Karrikinolide2 (KAR_2_)] are potent bioactive compounds from plant-derived smoke [12,13,14]. SW and KAR_1_ have been reported to stimulate the germination of seeds of several plant species with poor germination or with dormant seeds [10,15,16]. The smoke-derived biostimulants are highly effective and the single treatments have been shown to release the dormancy in lettuce seeds [10,17]. The possible mechanism by which this is achieved may be due to the substitution for red light by KAR_1_ via the interconversion of red light-absorbing (Pr) and far-red light-absorbing (Pfr) forms [10,18]. Thus, biostimulants prepared from plant-derived smoke are very promising for enhancing the germination of dormant seeds, such as celery seeds that require light for their germination.

The stimulus for a breakdown of celery endosperm emanates from the embryo in response to light [3,4]. It has been shown that dormancy in celery seeds was broken by a combination of plant-derived smoke and BA and gibberellins GA_4_ + GA_7_ [19]. They suggested that smoke extracts act similarly to cytokinins by enhancing gibberellin activity in the celery seed. However, no studies are available on how SW and KAR_1_ affect auxins in celery and how soaking affects in vivo KAR_1_ levels after the SW and KAR_1_ treatments of celery seeds.

In the present study, we investigated the effects of SW and KAR_1_ treatments on celery seed germination and dormancy release through direct application and seed soaking. Furthermore, we measured the dynamics of KAR_1_ content in KAR_1_- and SW-treated celery seeds to elucidate its possible mode of action in promoting seed germination and mitigating dormancy. Knowing how KAR_1_ is absorbed and translocated within plant systems might shed light on its biological functions and potential applications in seed germination and dormancy regulation. We also evaluated the impact of these treatments on the levels of auxin and cytokinins during seed germination. The findings from this study could improve the germination of morphologically dormant celery seeds and contribute to a broader understanding of how smoke-derived biostimulants can be harnessed to sustain crop yield. The insights gained from this study will deepen our understanding of advancing agricultural practices and addressing challenges related to seed dormancy in celery.

## 2. Results

### 2.1. Seed Germination of Celery

**Figure 1:**
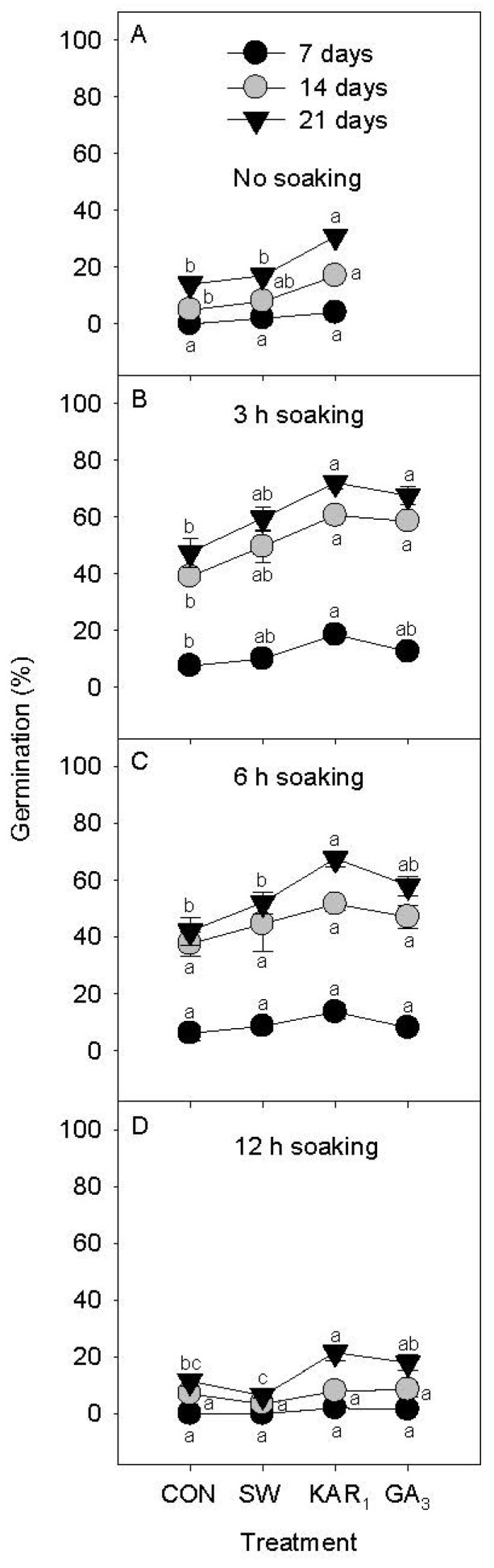
Effect of different soaking periods [(**A**) no soaking, (**B**) 3 h soaking, (**C**) 6 h soaking and (**D**) 12 h soaking] of smoke-water (SW 1:2500 *v*/*v*), Karrikinolide1 (KAR_1_ 10^−7^ M) and gibberellic acid (GA_3_ 10^−7^ M) on seed germination of celery under 14 h light and 10 h dark conditions at 20 °C (*n* = 3). Symbols (±SE) of each soaking period with different letter(s) are significantly different according to Tukey’s test (*p* < 0.05). CON = control treatment.

In non-seed soaking treatments, SW and KAR_1_ solutions were applied by placing the celery seeds on filter paper and subjecting them to a 21-day incubation period at 20 °C on a Jacobsen germination table as detailed earlier. On the 7th day, SW- and KAR_1_-treated seeds showed 2% and 4% germination, respectively. However, no seed germination was recorded in the control (Figure 1A). On the 14th day, 8% and 16.7% seed germination were obtained in SW- and KAR_1_-treated seeds, respectively. These results showed a progressive increase in percentage germination compared to the control, which showed only 4.7% germination. Furthermore, the results revealed improvement in celery seed germination and dormancy release with the application of KAR_1_, which exhibited significantly greater germination (30.7%) compared to the SW (17.2%) and control (14.7%) treatments at day 21 (Figure 1A).

KAR_1_ and SW treatments were compared with a positive control GA_3_ (10^−7^ M) and water control in the seed soaking experiment. In the 3 h soaking treatment, at day 21, 59.5%, 72.0%, and 67.5% germination were achieved in seeds treated with SW, KAR_1_, and GA_3_, respectively. At the same time, water control obtained 47.5% germination. KAR_1_ and GA_3_ results were significantly different from the control (Figure 1B). In the 6 h soaking treatment, at day 21, there was a slight decline in seed germination percentages for SW, KAR_1_, and GA_3_ treatments. The lowest germination percentage was recorded in water control (Figure 1C). In the 12 h soaking treatment, at day 21, seed germination reduced dramatically in all treatments, indicating a detrimental effect of prolonged soaking on germination (Figure 1D).

### 2.2. KAR_1_ Content in Celery Seeds

**Figure 2:**
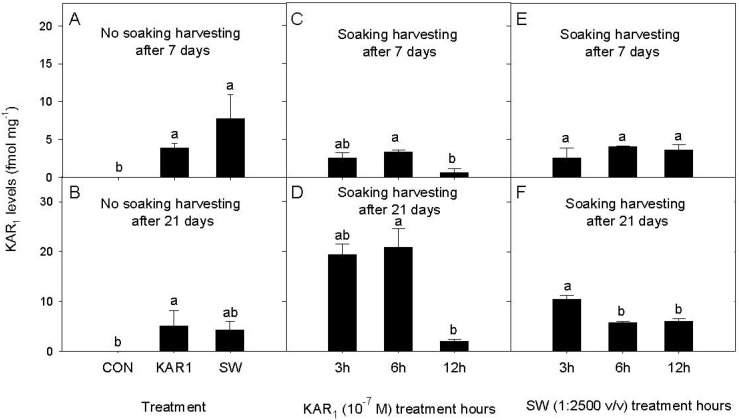
KAR_1_ levels in KAR_1_−, and SW−, treated celery seeds in no soaking [(**A**) no soaking harvesting after 7 days and (**B**) no soaking harvesting after 21 days] and soaking treatments [KAR_1_ soaking treatment− (**C**) harvesting after 7 days and (**D**) harvesting after 21 days; SW soaking treatment− (**E**) harvesting after 7 days and (**F**) harvesting after 21 days] after 7 and 21 days of harvesting. Bars (± SE) of each figure with different letter(s) are significantly different according to Tukey’s test (*p* < 0.05). CON = control treatment.

The results showed noteworthy trends in KAR_1_ content in celery seeds under different treatment conditions. When seeds were subjected to direct application of KAR_1_ and SW (without soaking), distinct levels of KAR_1_ were detected in the seeds harvested after 7 days (Figure 2A). Interestingly, the levels of KAR_1_ were significantly higher in seeds treated with SW (7.73 fmol mg^−1^) as compared to those treated with KAR_1_ alone (3.93 fmol mg^−1^) (Figure 2A). However, the levels of KAR_1_ in KAR_1_- and SW-treated seeds harvested after 21 days were 5.72 and 4.60 fmol mg^−1^, respectively (Figure 2B). These results were significantly different from the control.

In the soaking treatments, levels of KAR_1_ in KAR_1_-treated seeds harvested after 7 days varied depending on the duration of soaking (Figure 2C). After 3, 6, and 12 h of soaking, the levels of KAR_1_ were 2.53, 3.34, and 0.57 fmol mg^−1^, respectively (Figure 2C). Interestingly, after 21 days, the levels of KAR_1_ increased substantially to 19.3, 20.9, and 2.04 fmol mg^−1^ in seeds soaked for 3, 6, and 12 h, respectively (Figure 2D). The lowest level of KAR_1_ was detected for the 12 h soaking treatment, which was significantly different from 3 and 6 h soaking treatments. Similarly, in SW-treated seeds, the levels of KAR_1_ in seeds harvested after 7 days varied with soaking duration (Figure 2E). These KAR_1_ levels were 2.61, 4.05, and 3.66 fmol mg^−1^ after 3, 6, and 12 h of soaking, respectively (Figure 2E). However, there was no significant difference between the treatments, whereas, after 21 days, the levels of KAR_1_ increased to 10.0, 5.74, and 6.01 fmol mg^−1^ in seeds soaked for 3, 6, and 12 h, respectively. The highest level of KAR_1_ was recorded for 3 h soaking treatment, which was significantly different from the 6 and 12 h soaking treatments of SW (Figure 2F).

### 2.3. Effect of Different Soaking Periods of SW, KAR_1_, and GA_3_ on the Levels of Auxins

**Figure 3:**
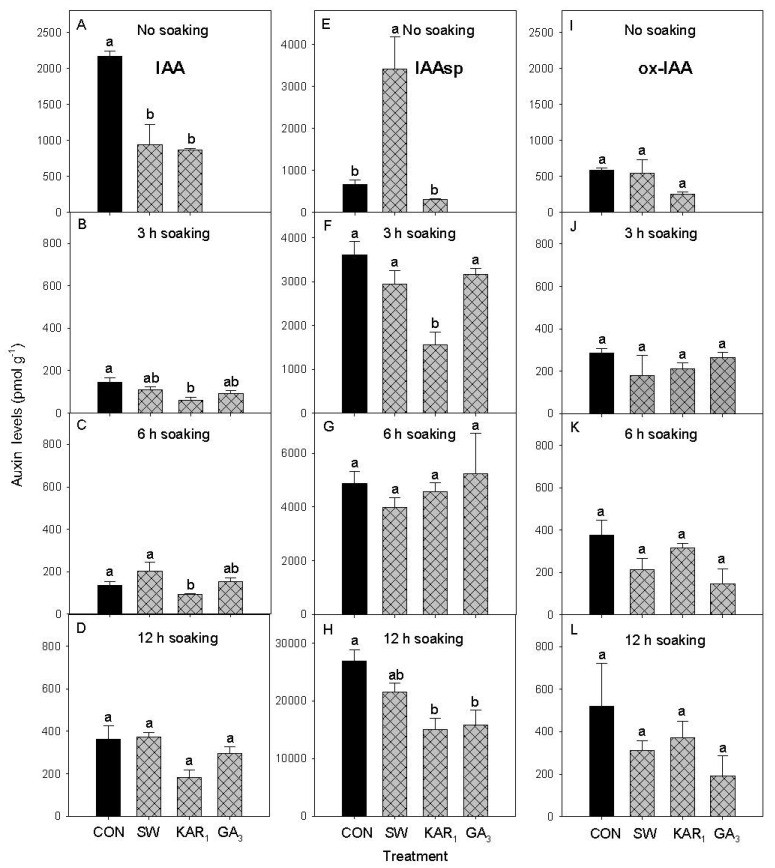
Effect of different soaking periods of smoke-water (SW 1:2500 *v*/*v*), Karrikinolide1 (KAR_1_ 10^−7^ M), and gibberellic acid (GA_3_ 10^−7^ M) on the levels of aromatic indole-3-acetic acid (IAA) [(**A**) No soaking, (**B**) 3 h soaking, (**C**) 6 h soaking and (**D**) 12 h soaking], 2-oxindole-3-acetic acid (ox-IAA) [(**E**) No soaking, (**F**) 3 h soaking, (**G**) 6 h soaking and (**H**) 12 h soaking], and indole-3-acetyl aspartic acid (IAAsp) [(**I**) No soaking, (**J**) 3 h soaking, (**K**) 6 h soaking and (**L**) 12 h soaking] auxins in 7-day-germinated celery seeds under 14 h light and 10 h dark conditions at 20 °C (*n* = 3). Bars (±SE) of each soaking period and auxin with different letter(s) are significantly different according to Tukey’s test (*p* < 0.05). CON = control treatment.

In seeds without soaking treatment, the levels of IAA were significantly higher in the water control (2169 pmol g^−1^) as compared to the seeds treated with SW (935 pmol g^−1^) and KAR_1_ (864 pmol g^−1^) (Figure 3A). The IAA levels in control were 2.51 and 2.32 times higher than KAR_1_ and SW. The levels of IAAsp were significantly higher in SW (3422 pmol g^−1^) compared to water control (656 pmol g^−1^) and KAR_1_ (305 pmol g^−1^) (Figure 3E).

The level of IAA after 3 h soaking treatment was significantly higher in control (145 pmol g^−1^, 2.47 times higher) as compared to KAR_1_ treatment (58 pmol g^−1^) (Figure 3B). However, no significant differences were observed in IAA levels when compared to SW and GA_3_ (positive control). A similar trend was observed in the level of IAAsp, where the level of IAAsp in control (3610 pmol g^−1^) was 2.2 times higher as compared to KAR_1_ (1569 pmol g^−1^) (Figure 3F). After 6 h of soaking treatment, the level of IAA in control (236 pmol g^−1^) was 2.5 times higher as compared to KAR_1_ (91 pmol g^−1^) (Figure 3C). No significant differences were observed in IAA levels when compared to SW and GA_3_ treatments. After 12 h of soaking treatment, the level of IAA in control (364 pmol g^−1^) was 2.0 and 1.22 times higher as compared to KAR_1_ (181 pmol g^−1^) and GA_3_ (297 pmol g^−1^), respectively. However, these differences were non-significant (Figure 3D). The level of IAAsp in water control (26,913 pmol g^−1^) was 1.78 and 1.70 times significantly higher compared to KAR_1-_treated seeds (15,103 pmol g^−1^) and GA_3_-treated seeds (15,812.79 pmol g^−1^), respectively, in 12 h soaking treatment (Figure 3H). No significant differences were found in ox-IAA levels, both without soaking and soaking treatments (Figure 3I–L). This suggests that ox-IAA levels were not influenced by the treatments evaluated in the present study.

### 2.4. Effect of Different Soaking Periods of SW, KAR_1_, and GA_3_ on the Levels of Cytokinins

**Figure 4:**
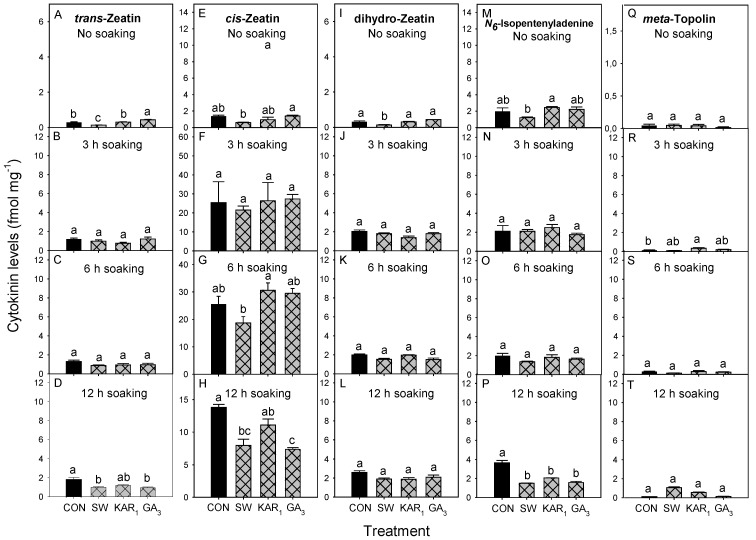
Effect of different soaking periods of smoke-water (SW 1:2500 *v*/*v*), Karrikinolide1 (KAR_1_ 10^−7^ M), and gibberellic acid (GA_3_ 10^−7^ M) on the levels cytokinins [*trans-*Zeatin (**A**) No soaking, (**B**) 3 h soaking, (**C**) 6 h soaking and (**D**) 12 h soaking; *cis-*Zeatin (**E**) No soaking, (**F**) 3 h soaking, (**G**) 6 h soaking and (**H**) 12 h soaking; dihydro-Zeatin (**I**) No soaking, (**J**) 3 h soaking, (**K**) 6 h soaking and (**L**) 12 h soaking; N6-Isopentenyladenine (**M**) No soaking, (**N**) 3 h soaking, (**O**) 6 h soaking and (**P**) 12 h soaking; *meta*-topolin (**Q**) No soaking, (**R**) 3 h soaking, (**S**) 6 h soaking and (**T**) 12 h soaking] in 7-day-germinated celery seeds under 14 h light and 10 h dark conditions at 20 °C (*n* = 3). Bars (±SE) of each soaking period and auxin with different letter(s) are significantly different according to Tukey’s test (*p* < 0.05). CON = control treatment.

In the present study, N6-Isopentenyladenine was the predominant cytokinin found in 7 day-germinated celery seeds in without soaking treatments (Figure 4M), and in soaking treatments *cis*-Zeatin was predominant (Figure 4F,G,H). After 7 days of celery seed germination in non-soaking treatment, the levels of *trans-Zeatin* were significantly high in GA_3_-treated seeds (0.43 fmol mg^−1^) (Figure 4A), other treatments (KAR_1_, SW, and CON) exhibited lower *trans-Zeatin* levels, with SW showing the least amount (0.13 fmol mg^−1^). Whereas, the levels of *cis-Zeatin*, dihydro-Zeatin, N6-Isopentenyladenine and *meta*-topolin after 7 days were non-significant in all treatments (Figure 4E,I,M,Q). On the other hand, in soaking treatments the differences in levels of all cytokinins after 7 days were non-significant for all treatments, except for N6-Isopentenyladenine in the water controls of 12 h soaking (3.65 fmol mg^−1^) treatments (Figure 4P).

**Figure 5:**
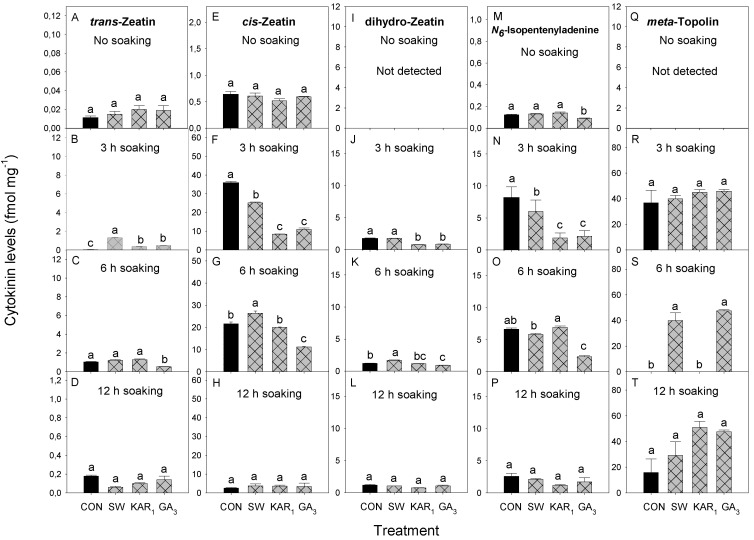
Effect of different soaking periods of smoke-water (SW 1:2500 *v*/*v*), Karrikinolide1 (KAR_1_ 10^−7^ M), and gibberellic acid (GA_3_ 10^−7^ M) on the levels cytokinins [*trans-*Zeatin (**A**) No soaking, (**B**) 3 h soaking, (**C**) 6 h soaking and (**D**) 12 h soaking; *cis-*Zeatin (**E**) No soaking, (**F**) 3 h soaking, (**G**) 6 h soaking and (**H**) 12 h soaking; dihydro-Zeatin (**I**) No soaking, (**J**) 3 h soaking, (**K**) 6 h soaking and (**L**) 12 h soaking; N6-Isopentenyladenine (**M**) No soaking, (**N**) 3 h soaking, (**O**) 6 h soaking and (**P**) 12 h soaking; *meta*-topolin (**Q**) No soaking, (**R**) 3 h soaking, (**S**) 6 h soaking and (**T**) 12 h soaking] in 21-day-germinated celery seeds under 14 h light and 10 h dark conditions at 20 °C (*n* = 3). Bars (±SE) of each soaking period and auxin with different letter(s) are significantly different according to Tukey’s test (*p* < 0.05). CON = control treatment.

After 21 days of celery seed germination, *cis*-Zeatin was found to be predominant cytokinin in without soaking treatment (Figure 5E) and in soaking treatment *meta*-Topolins were the predominant cytokinins (Figure 5R–T). After 21 days, in without soaking treatment, the levels of N6-Isopentenyladenine were significantly low in GA_3_-treated seeds (0.091 fmol mg^−1^), as compared to KAR_1_ (0.14 fmol mg^−1^), SW (0.13 fmol mg^−1^) and water control (0.12 fmol mg^−1^) (Figure 5M), whereas the levels of *trans-Zeatin* and *cis-Zeatin* were non-significant (Figure 5A,E), and dihydro-Zeatin and *meta*-Topolin were not detected (Figure 5I,Q).

In the soaking treatments after 21 days, the levels of all cytokinins were non-significant for all treatments, except for the levels of *trans*-Zeatin in 3 h soaking (highest levels in SW-treated seeds, 1.47 fmol mg^−1^) (Figure 5B), *cis*-Zeatin in 3 h soaking (highest levels in water control, 35.7 fmol mg^−1^) (Figure 5F) and 6h soaking (highest levels in SW-treated seeds, 26.6 fmol mg^−1^) (Figure 5G), and N6-Isopentenyladenine in 3 h soaking (highest levels in water control, 6.62 fmol mg^−1^) (Figure 5N).

The levels of *meta*-topolins were non-significant except for the 6 h soaking treatment, where SW- (39.9 fmol mg^−1^) and GA_3_-treated (47.5 fmol mg^−1^) seeds showed significantly higher contents of *meta*-topolins compared to the KAR_1_-treated and water control seeds (Figure 5S).

## 3. Discussion

Overall, the results indicate that both SW and KAR_1_ treatments successfully promote celery dormancy release and seed germination, whether administered directly or through seed soaking. The seed soaking treatment showed that soaking celery seeds in KAR_1_ for 3 h was best for effectively breaking the morphological dormancy and remarkably improving the germination rate followed by GA_3_ and SW. The ideal seed soaking time is important because longer soaking times have a negative impact on germination. When seeds are soaked for a longer duration, several coumarin-based chemicals found in celery seeds may be leached from the seed coat, preventing them from germinating [6,7,20,21]. As celery seeds are difficult to germinate, priming or soaking of seeds has been the subject of extensive research [20,22,23,24]. However, it has been observed that different varieties and even seed lots of the same variety respond differently to a priming treatment, making celery priming less successful [24,25]. Hence, research on the mechanisms underlying celery seed priming or soaking has yet to be undertaken in detail.

The transportation mechanisms of Karrikins, specifically KAR_1_, in plant systems represent a critical area of biostimulant research, which is largely unexplored. In this study, we investigated KAR_1_ content in celery seeds treated with both KAR_1_ and SW to shed light on the dynamics of Karrikin and its implications for seed germination. The sustained presence of KAR_1_ levels in the seeds treated with both SW and KAR_1_ suggests that it may have prolonged effects on dormancy release and seed germination. Additionally, different soaking durations play a crucial role in modulating KAR_1_ content, with prolonged soaking resulting in higher levels of KAR_1_ in seeds. KAR_1_ is likely to play a significant role in the germination process of celery seeds, as it is highly active at very low concentrations, water soluble, thermostable, and long lasting in solution [26]. Furthermore, the variations in the levels of KAR_1_ in celery seeds over time suggest that highly complex mechanisms control its absorption, distribution, and metabolism, which require further investigation. This will help to extend our knowledge of plant signaling systems and utilization of Karrikins to alleviate dormancy and enhance seed germination.

The effect of different soaking periods of SW, KAR_1_, and GA_3_ on the levels of indole-3-acetic acid (IAA) and two auxin catabolites, 2-oxindole-3-acetic acid (ox-IAA), and indole-3-acetyl aspartic acid conjugate (IAAsp) were evaluated in germinated celery seeds after 7 days (Figure 3). The level of active auxin is regulated by the formation of irreversible catabolites, the oxidized form of IAA, 2-oxindole-3-acetic acid (oxIAA), and the amide-linked IAA conjugates to aspartate and glutamate (IAA-Asp and IAA-Glu) [27]. On day 7, seed germination was observed in the SW and KAR_1_ treatments, but no seed germination was recorded in the water control. This highlights the potential efficacy of the SW and KAR_1_ in promoting germination, which is why the hormones were evaluated on day 7. GA_3_ (10^−7^ M) was considered as a positive control in soaking treatment. Out of all the auxins evaluated, only three forms were detected, indole-3-acetic acid (IAA), indole-3-acetyl aspartic acid (IAAsp), and 2-oxindole-3-acetic acid (ox-IAA) (Figure 3A–L). It is important to note that, in comparison to the conjugate IAAsp, oxIAA was found to be a minor IAA catabolite in the present study. This implies that IAA conjugation to Asp is the major catabolic pathway regulating IAA levels in celery seeds during germination. It was also noted that soaking treatments reduced the levels of IAA compared to those without soaking treatments. The levels of IAA in no soaking treatments were 14.9-, 8.57-, and 14.8-fold higher in control, SW, and KAR_1_, respectively, compared to 3 h soaking (Figure 3A,B). Similarly, lower levels of IAA were detected for 6 and 12 h of soaking treatments compared to no soaking treatment. It has been reported that IAA content in celery fruits rapidly declined approximately 7-fold upon imbibition [28].

The present study demonstrates the effects of different soaking treatments on IAA levels in celery seeds. A negative correlation was observed in the levels of IAA and seed germination in all treatments. The correlations were found to be −0.88, −0.97, −0.91, and −0.93 for no soaking, 3, 6, and 12 h soaking treatments (Table S1), respectively, suggesting that IAA is involved in inhibiting celery seed germination. Other than ABA, IAA is an additional plant hormone that has been found to cause seed dormancy [29]. Studies have also reported that auxins have an inhibitory role in seed germination and the IAA level in mature seeds appeared to be linked to dormancy [30]. It has been reported that treatment of wheat seeds with exogenous IAA or IAA precursors (e.g., tryptophan) inhibited germination, while IAA biosynthesis inhibitors or antagonists of IAA overcame the germination inhibitory effects of IAA or their precursors [31]. Exogenous auxin (IAA) treatment also inhibited Arabidopsis seed germination under salt stress conditions [32]. IAA inhibited Arabidopsis seed germination in an ABA-dependent manner [33]. However, the underlying mode of action of auxin function in seed dormancy is still unclear. It is important to note that no soaking treatment exhibited the highest negative correlation of −0.88, coupled with the lowest seed germination observed on the 7th day along with the highest IAA levels, suggesting that the soaking treatment also assisted in reducing the levels of IAA. However, 12 h soaking treatment was found to be inhibitory to seed germination. Furthermore, KAR_1_ treatment reduced the levels of IAA in all treatments; this implies that the synthesis, accumulation, or activity of IAA within the celery seeds may be disrupted or inhibited by KAR_1_ treatment. Since IAA has been linked to inhibition of the germination process and induction of seed dormancy [31,34], lowering its levels via biostimulants such as KAR_1_ may help to reduce dormancy and improve germination efficiency. Reducing IAA levels by KAR_1_ treatment highlights the role of modifying hormone interactions and influencing physiological processes during seed germination. However, it is crucial to carry out further research to determine the processes.

In the present study, the differences in levels of all cytokinins were non-significant for all treatments except for N6-Isopentenyladenine. The levels of N6-Isopentenyladenine were significantly higher in the KAR_1_ treated seeds compared to SW of no soaking treatments of seeds analysed after 7 days of germination (Figure 4M) and in the control of 3 h soaking treatment of seeds analysed after 21 days of germination (Figure 5N) compared to other treatments. In these treatments, no-to-very-low germination was observed, which could be attributed to the presence of high amounts of N6-Isopentenyladenine. In the case of *Paris polyphylla* (love apple), it has been shown that N6-Isopentenyladenine is negatively correlated with the germination of seeds [35]. However, further research is needed to confirm and elucidate the specific mechanisms by which N6-Isopentenyladenine affects seed germination in celery.

The *cis*-Zeatin has been reported to be inactive or has a weak biological activity, however, some studies suggest a role of *cis*-Zeatin in dormancy and seed germination [36,37,38]. In Arabidopsis, *cis*-Zeatin-type cytokinins are prevalent in the developmental stages associated with limited growth [37]. It is also reported that *cis*-Zeatin is involved in establishing dormancy in mature *Lolium rigidum* (annual ryegrass) seeds [39]. The findings of the present study indicate that cytokinins may not play a central role in controlling celery seed germination and dormancy under normal temperature and light conditions. It has been proposed that GA has a more central role than cytokinins in controlling celery seed germination under high temperature treatment [40,41]. It has also been reported that naturally occurring cytokinins, zeatin, and zeatin riboside, showed no activity in celery seeds [42]. Cytokinins may also inhibit or delay the emergence of the radicle within the seed [43]. However, further research is required to thoroughly understand the precise mode of action by which these cytokinins affect the seed physiology of celery and those of other plant species.

## 4. Materials and Methods

### 4.1. Plant Material

Celery (*Apium graveolens* L.) seeds (cultivar Oderdorfer, accession No. 09H1000051, variety rapaceum), which originated from the former German Democratic Republic were procured from the Czech national collection of plant genetic resources at the Crop Research Institute Praha-Ruzyně, Czech Republic. They were stored in the dark at 4 °C in an opaque bag until used.

### 4.2. Smoke Compounds and Chemicals

Smoke-water and Karrikinolide1 solutions were prepared according to previously described methods [10,11,12,13]. All the chemicals used in the study were of analytical grade.

### 4.3. Experimental Site

The germination experiments were carried out at the Crop Research Institute, Olomouc and the analytical experiments were performed at the Laboratory of Growth Regulators, Palacký University Olomouc, Czech Republic.

### 4.4. Germination Conditions

For the preliminary (first trial without soaking) experiment, the celery seeds were tested with SW and KAR_1_ for the germination percentage. The seeds were treated with 70% ethanol for 30 sec and then washed thoroughly with sterile distilled water for germination. Polystyrene Petri dishes (90 mm) were lined with two sheets of standard laboratory filter paper with small cavities marked with a punch machine. The filter paper was moistened once with 4.2 mL of the different test solutions, SW (1:2500 *v*/*v*), KAR_1_ (10^−7^ M), and sterile distilled water, which was used as a control. The Petri dishes were then placed in a room equipped with a Jacobsen’s germination table at 20 ± 1 °C for 21 d with 10 h light and 14 h dark conditions. The seeds were considered germinated when the radicle was at least 2 mm long. The seeds were moistened with sterile distilled water when needed. The readings were recorded on the 7th, 14th, and 21st day. Four replicates with 50 seeds each were used for the germination of celery seeds. The experiment was repeated thrice.

For the soaking experiment, the seeds were tested with SW (1:2500 *v*/*v*), KAR_1_ (10^−7^ M), and GA_3_ (10^−7^ M) for germination by using the seed soaking method. The seeds were soaked in the test solutions for 3, 6, and 12 h and were air-dried. Sterile distilled water (4.2 mL) was pipetted onto the two layers of filter paper in the Petri dishes. The Petri dishes were then placed in a growth room at 20 ± 1 °C for 21 d with 10 h light and 14 h dark conditions. The seeds were moistened with sterile distilled water when needed. The experiment was repeated thrice.

### 4.5. Estimation of KAR_1_ in Plant Sample

After 7–21 days of imbibition, celery seeds in KAR_1_ and SW were washed twice to remove adhering smoke compounds from the surface of the seeds. Subsequently, the seeds were collected in sterile plastic falcon tubes and the tubes were immediately immersed in liquid nitrogen to stop any possible degradation processes in the plant samples. The falcon tubes were transferred to a −80 °C freezer for storage until analyzed. The samples were homogenized in a sterile mortar and pestle using liquid nitrogen. The homogenized samples were weighed (approximately 10 mg per sample) and were stored in a deep freezer at −80 °C until further use. Each biological sample had 3 technical replicates for higher accuracy. KAR_1_ samples were extracted in 1 mL ice-cold acidified 10% methanol with 10 pmol of stable isotopically labelled internal standard of karrikin. Samples were purified using solid phase extraction and analyzed by an Acquity UPLC^®^ I-Class sytem (Waters, Milford, MA, USA) combined with a Xevo™ TQ-S triple quadrupole mass spectrometer (Waters, Manchester, UK) and an Acquity UPLC^®^ BEH C18 reversed-phase column (1.7 µm, 2.1 × 50 mm, Waters) [44].

### 4.6. Estimation of Phytohormones

The treated seed samples were processed for auxin and cytokinin analyses. The homogenized samples were weighed (approximately 3 mg per sample) and were stored in a deep freezer (–80 °C) until further use. Each biological sample had three technical replicates for higher accuracy. IAA and CK samples were extracted in 1 mL of modified Bieleski buffer (methanol/water/formic acid 15/4/1 *v*/*v*/*v*) with an internal standard of stable isotopically labelled internal standard (0.2 pmol per sample of CK bases, ribosides, 7- and 9-glucosides, and 0.5 pmol per sample of CK O-glucosides and nucleotides, and 1.0 pmol of IAA and IAA-conjugates) to the determination of concentration. Samples were purified using a pipette tip solid-phase purification [45] and analyzed by ultraperformance liquid chromatography coupled to a triple quadrupole mass spectrometer (CK analysis: Acquity UPLC^®^ I-class System (Waters, Milford, MA, USA) equipped with Acquity UPLCW BEH C18 column (1.7 μm, 2.1 × 150 mm, Waters) and linked to Xevo TQ-S (Waters, Manchester, UK). For IAA analysis, the HPLC system 1260 Infinity II (Agilent Technologies, Santa Clara, CA, USA) was equipped with a Kinetex C18 column (1.7 µm, 2.1 × 50 mm, Phenomenex) and linked to a 6495 Triple Quad detector (Agilent Technologies, Santa Clara, CA, USA) [46,47].

The levels of natural isoprenoid cytokinins comprising the 2-C-methyl-D-erythritol 4-phosphate (MEP) pathway (plastid)-derived cytokinins viz., *t*Z-type cytokinins (*trans-*Zeatin), DHZ-type cytokinins (dihydro-Zeatin) and iP-type cytokinins (N6-(2-isopentenyl)adenine-type), the mevalonate (MVA) pathway (cytosol)-derived cytokinin- *c*Z-type cytokinins (*cis-*Zeatin), and aromatic cytokinins- *meta*-Topolin (*m*T) were determined in 7- and 21-day-old non-soaked and soaked celery seeds using UHPLC-MS/MS. These time points (7- and 21-day-old) were selected to study the role of cytokinins in early growth and late developmental processes (involved in nutrient mobilization and delayed senescence). Furthermore, the levels of aromatic auxins viz., indole-3-acetic acid (IAA), 2-oxindole-3-acetic acid (ox-IAA), and indole-3-acetyl aspartic acid (IAAsp) were evaluated in 7-day-old non-soaked and soaked celery seeds. In the case of auxins, their levels were analyzed only in 7-day-old non-soaked and soaked celery seeds to study the peak activity and their relevance during early seedling development as they are involved in the promotion of dormancy.

### 4.7. Statistical Analysis

Germination data were arcsine-transformed prior to statistical analysis. The data were subjected to one-way analysis of variance (ANOVA) and significant differences between treatments of germination assays and hormone analysis were determined using Tukey’s test at a 95% confidence interval (*p* < 0.05) [48]. GenStat^®^ (Version 23.1, Rothamsted Research, Harpenden, UK) statistical package was used for the data analysis.

## 5. Conclusions

Smoke-water (SW) and Karrikinolide1 (KAR_1_) growth-promoting substances were investigated to break the morphological dormancy of the celery cultivar (*Apium graveolens* L.). The direct application of SW and KAR_1_ significantly increased the germination percentage of celery seeds compared to untreated controls. Furthermore, seed soaking treatments showed that KAR_1_-treated seeds soaked for 3 h were most effective in improving celery seed germination, which outperformed positive control gibberellic acid (GA_3_) treatment. The measurement of KAR_1_ in celery seeds provides insight into its distribution within seeds over time, indicating its effects on dormancy alleviation and germination. Furthermore, analysis of auxin (particularly IAA) levels revealed a negative correlation between IAA levels and seed germination. KAR_1_ treatment reduced IAA levels, suggesting its role in alleviating dormancy and improving seed germination. SW and KAR_1_ did not significantly change cytokinin levels during celery germination, except N6-Isopentenyladenine, which was generally lower than water control. The present study advances our understanding of how plant-based hormone responses are influenced by bioactive compounds from plant-derived smoke, providing valuable information for improving germination efficiency in celery.

## Data Availability

The datasets used and/or analyzed during the current study are available from the corresponding author on reasonable request.

## Supplementary Materials

The following supporting information can be downloaded at: https://www.mdpi.com/article/10.3390/plants13152096/s1, Table S1: A Bioactive Compound Karrikinolide1 (KAR1) from Smoke Improves the Germination of Morphologically Dormant *Apium graveolens* L. Seeds by Reducing Indole-3-Acetic Acid (IAA) Levels.
